# Draft genome sequences of 109 gut bacteria isolated from colonoscopy samples of individuals with and without cystic fibrosis

**DOI:** 10.1128/mra.01114-25

**Published:** 2026-03-12

**Authors:** Sarvesh V. Surve, Rebecca A. Valls, George A. O'Toole

**Affiliations:** 1 Department of Microbiology and Immunology, Geisel School of Medicine at Dartmouth, Hanover, New Hampshire, USA; Loyola University Chicago, Chicago, Illinois, USA

**Keywords:** cystic fibrosis, culturomics, intestine, microbiome

## Abstract

We report draft genome sequences of gut bacterial isolates recovered from colonoscopy samples obtained from individuals with and without cystic fibrosis. These genomes expand the representation of cultured gut microbes from distinct colonic regions and provide a resource for studying microbial diversity, adaptation, and host-microbe interactions in health and disease.

## ANNOUNCEMENT

The human gut microbiome is a critical determinant of host health, and its disruption has been linked to gastrointestinal and systemic diseases (1, 2). For cystic fibrosis (CF), intestinal dysbiosis is well documented and may contribute to altered nutrient absorption and inflammation (3–5). To better understand gut microbial diversity in persons with CF (pwCF), we conducted a combined culture-based and sequencing-based analysis of colonoscopy samples. Here, we present draft genome sequences of bacterial isolates cultured from ascending, transverse, and descending colon samples collected from four pwCF, two heterozygotes (one CF and one non-CF allele), and four non-CF adults (Table 1). One heterozygous individual (GIEHZ101) provided additional samples from the esophagus and small intestine.

**TABLE 1:** Genome data for individual strains

Strain	Assembly accession	SR sample	Organism name	WGS project accession	Coverage	Contigs	Size	%GC	Contig N50	Location	Patient	Genotype	Media	Status	Type
GP0003	GCA_030546315.1	SRS18318715	*Lactobacillus paragasseri*	JAUONS01	677.99	25	2,047,530	34.80	237,129	Esophagus	GIEHZ101	Heterozygous	BBE	Anaerobic	Lumen
GP0011	GCA_030546305.1	SRS18318716	*Streptococcus* sp. GP0011	JAUONR01	828.09	20	2,109,804	40.93	274,222	Esophagus	GIEHZ101	Heterozygous	BA + 100 μg/mL gentamicin	Anaerobic	Lumen
GP0012	GCA_030546285.1	SRS18318727	*Streptococcus oralis*	JAUONQ01	708.05	24	2,049,680	41.03	206,611	Esophagus	GIEHZ101	Heterozygous	BA + 100 μg/mL gentamicin	Anaerobic	Lumen
GP0025	GCA_030546245.1	SRS18318738	*Streptococcus oralis*	JAUONP01	753.26	24	2,071,804	40.99	206,761	Esophagus	GIEHZ101	Heterozygous	BA + 100 μg/mL gentamicin	Anaerobic	Lumen
GP0046	GCA_030541705.1	SRS18318749	*Ligilactobacillus animalis*	JAUNZL01	876.09	43	1,922,642	41.00	148,838	Small bowel	GIEHZ101	Heterozygous	BBE	Anaerobic	Lumen
GP0053	GCA_030546225.1	SRS18318760	*Streptococcus oralis*	JAUONO01	921.12	24	2,073,577	40.99	206,791	Small bowel	GIEHZ101	Heterozygous	BA + 100 μg/mL gentamicin	Anaerobic	Lumen
GP0067	GCA_031119655.1	SRS18256170	*Phocaeicola vulgatus*	JAVHZY01	362.00	69	5,030,823	42.19	163,280	Descending	GIEHZ101	Heterozygous	BA + 100 μg/mL gentamicin	Anaerobic	Mucosa
GP0068	GCA_030546205.1	SRS18318764	*Phocaeicola vulgatus*	JAUONN01	289.80	69	4,965,451	42.20	163,998	Descending	GIEHZ101	Heterozygous	BA + 100 μg/mL gentamicin	Anaerobic	Mucosa
GP0074	GCA_030546235.1	SRS18318765	*Clostridium perfringens*	JAUONM01	463.29	50	3,484,746	28.00	345,113	Descending	GIEHZ101	Heterozygous	BBE	Anaerobic	Lumen
GP0081	GCA_030541685.1	SRS18318766	*Phocaeicola vulgatus*	JAUNZK01	303.13	69	5,033,962	42.19	164,271	Descending	GIEHZ101	Heterozygous	BA + 100 μg/mL gentamicin	Anaerobic	Mucosa
GP0119	GCA_030546185.1	SRS18318767	*Bacteroides caccae*	JAUONL01	238.78	81	5,443,876	42.93	209,659	Descending	GIEHV107	Non-CF	BA + 100 μg/mL gentamicin	Anaerobic	Lumen
GP0120	GCA_030546165.1	SRS18318717	*Bacteroides caccae*	JAUONK01	271.34	84	5,439,777	42.92	209,873	Descending	GIEHV107	Non-CF	BA + 100 μg/mL gentamicin	Anaerobic	Lumen
GP0122	GCA_030546125.1	SRS18318718	*Bacteroides caccae*	JAUONJ01	274.76	80	5,476,814	42.90	340,352	Descending	GIEHV107	Non-CF	BA + 100 μg/mL gentamicin	Anaerobic	Lumen
GP0136	GCA_023274715.1	SRR35288065	*Bacteroides fragilis*	JALXKF01	139.16	26	5,222,669	43.26	491,737	Ascending	GIEHZ100	Heterozygous	BBE + 100 μg/mL gentamicin	Anaerobic	Mucosa
GP0137	GCA_023274895.1	SRR35288064	*Bacteroides fragilis*	JALXKE01	28.05	34	5,222,451	43.26	369,120	Ascending	GIEHZ100	Heterozygous	BBE + 100 μg/mL gentamicin	Anaerobic	Lumen
GP0139	GCA_024357725.1	SRR35288053	*Bacteroides fragilis*	JANEWD01	638.06	25	5,223,094	43.26	491,737	Ascending	GIEHZ100	Heterozygous	BBE + 100 μg/mL gentamicin	Anaerobic	Mucosa
GP0153	GCA_024295025.1	SRR35288042	*Clostridium perfringens*	JANCNR01	259.00	18	3,374,812	28.12	2,112,539	Ascending	GIEHZ100	Heterozygous	BA + 100 μg/mL gentamicin	Anaerobic	Mucosa
GP0158	GCA_024296465.1	SRS13660907	*Klebsiella pneumoniae*	JANCNG01	531.21	62	5,537,862	57.14	391,040	Ascending	GIEHZ100	Heterozygous	McC	Aerobic	Mucosa
GP0162	GCA_024261365.1	SRR35288039	*Bacteroides fragilis*	JANCLQ01	61.81	31	5,248,611	43.22	398,716	Ascending	GIEHZ100	Heterozygous	BA	Anaerobic	Mucosa
GP0165	GCA_023274035.1	SRR35288038	*Escherichia coli*	JALXJY01	331.71	123	5,281,177	50.70	108,256	Ascending	GIEHZ100	Heterozygous	McC	Aerobic	Mucosa
GP0167	GCA_023275035.1	SRR35288037	*Bacteroides fragilis*	JALXJW01	37.32	34	5,222,185	43.26	407,866	Ascending	GIEHZ100	Heterozygous	BBE + 100 μg/mL gentamicin	Anaerobic	Lumen
GP0172	GCA_023276335.1	SRR35288036	*Bacteroides fragilis*	JALXKA01	328.07	27	5,223,195	43.26	491,737	Ascending	GIEHZ100	Heterozygous	BBE + 100 μg/mL gentamicin	Anaerobic	Lumen
GP0192	GCA_023274505.1	SRR35288035	*Escherichia coli*	JALXKB01	743.65	117	5,282,154	50.70	113,782	Ascending	GIEHZ100	Heterozygous	McC	Aerobic	Mucosa
GP0207	GCA_023274245.1	SRR35288034	*Bacteroides fragilis*	JALXJX01	65.49	38	5,222,481	43.26	369,120	Transverse	GIEHZ100	Heterozygous	BBE + 100 μg/mL gentamicin	Anaerobic	Mucosa
GP0208	GCA_024295045.1	SRR35288063	*Bacteroides fragilis*	JANCNO01	566.20	26	5,222,464	43.26	491,737	Transverse	GIEHZ100	Heterozygous	BBE + 100 μg/mL gentamicin	Anaerobic	Mucosa
GP0209	GCA_024295145.1	SRR35288062	*Bacteroides fragilis*	JANCLR01	96.45	32	5,223,726	43.26	398,716	Transverse	GIEHZ100	Heterozygous	BBE + 100 μg/mL gentamicin	Anaerobic	Lumen
GP0210	GCA_024128795.1	SRR35288061	*Bacteroides fragilis*	JAMYHA01	471.59	25	5,223,195	43.26	491,737	Transverse	GIEHZ100	Heterozygous	BBE + 100 μg/mL gentamicin	Anaerobic	Mucosa
GP0211	GCA_024294945.1	SRR35288060	*Bacteroides fragilis*	JANCNQ01	117.87	29	5,245,989	43.22	491,616	Transverse	GIEHZ100	Heterozygous	BBE + 100 μg/mL gentamicin	Anaerobic	Mucosa
GP0224	GCA_023894135.1	SRR35288059	*Bacteroides fragilis*	JAMWYN01	546.65	24	5,222,967	43.26	491,737	Transverse	GIEHZ100	Heterozygous	BA + 100 μg/mL gentamicin	Anaerobic	Lumen
GP0226	GCA_023958435.1	SRR35288058	*Bacteroides fragilis*	JAMXHY01	653.18	26	5,248,878	43.22	491,737	Transverse	GIEHZ100	Heterozygous	BA + 100 μg/mL gentamicin	Anaerobic	Mucosa
GP0234	GCA_030546135.1	SRS18318719	*Escherichia coli*	JAUONI01	311.75	119	5,060,842	50.55	185,941	Ascending	GIEHV103	Non-CF	BBE	Anaerobic	Lumen
GP0236	GCA_030546085.1	SRS18318720	*Escherichia coli*	JAUONH01	280.75	101	5,109,526	50.69	143,569	Ascending	GIEHV103	Non-CF	BBE	Anaerobic	Mucosa
GP0237	GCA_030546095.1	SRS18318721	*Enterococcus faecalis*	JAUONG01	522.49	46	2,989,804	37.27	222,965	Ascending	GIEHV103	Non-CF	BBE	Anaerobic	Lumen
GP0238	GCA_030546065.1	SRS18318722	*Enterococcus faecalis*	JAUONF01	562.83	46	2,990,024	37.27	222,965	Ascending	GIEHV103	Non-CF	BBE	Anaerobic	Mucosa
GP0247	GCA_030546045.1	SRS18318723	*Escherichia coli*	JAUONE01	336.01	120	5,060,233	50.55	171,587	Ascending	GIEHV103	Non-CF	BA + 100 μg/mL gentamicin	Anaerobic	Lumen
GP0281	GCA_030546025.1	SRS18318724	*Escherichia coli*	JAUOND01	327.42	113	5,058,319	50.55	192,308	Ascending	GIEHV103	Non-CF	BA + 100 μg/mL gentamicin	Anaerobic	Lumen
GP0282	GCA_030545975.1	SRS18318725	*Enterococcus gallinarum*	JAUONC01	360.62	44	3,266,419	40.38	194,676	Ascending	GIEHV103	Non-CF	BA + 100 μg/mL gentamicin	Anaerobic	Lumen
GP0284	GCA_030545985.1	SRS18318726	*Escherichia coli*	JAUONB01	211.86	115	5,059,852	50.55	177,605	Ascending	GIEHV103	Non-CF	BA + 100 μg/mL gentamicin	Anaerobic	Lumen
GP0305	GCA_030545965.1	SRS18318728	*Enterococcus faecalis*	JAUONA01	555.96	47	2,989,296	37.27	222,965	Transverse	GIEHV103	Non-CF	BBE	Anaerobic	Lumen
GP0306	GCA_030545945.1	SRS18318729	*Escherichia coli*	JAUOMZ01	311.81	112	5,064,224	50.55	171,587	Transverse	GIEHV103	Non-CF	BBE	Anaerobic	Lumen
GP0308	GCA_030545925.1	SRS18318730	*Enterococcus faecalis*	JAUOMY01	508.47	46	2,989,701	37.27	222,896	Transverse	GIEHV103	Non-CF	BBE	Anaerobic	Lumen
GP0319	GCA_030545895.1	SRS18318731	*Escherichia coli*	JAUOMX01	150.09	99	5,024,289	50.58	213,311	Transverse	GIEHV103	Non-CF	BA + 100 μg/mL gentamicin	Anaerobic	Lumen
GP0320	GCA_030545885.1	SRS18318732	*Escherichia coli*	JAUOMW01	319.33	114	5,061,766	50.55	171,587	Transverse	GIEHV103	Non-CF	BA + 100 μg/mL gentamicin	Anaerobic	Lumen
GP0322	GCA_030545865.1	SRS18318733	*Escherichia coli*	JAUOMV01	340.22	107	5,010,822	50.59	192,308	Transverse	GIEHV103	Non-CF	BA + 100 μg/mL gentamicin	Anaerobic	Mucosa
GP0351	GCA_030545845.1	SRS18318734	*Escherichia coli*	JAUOMU01	257.34	119	5,061,678	50.55	203,814	Transverse	GIEHV103	Non-CF	BA + 100 μg/mL gentamicin	Anaerobic	Lumen
GP0365	GCA_030545825.1	SRS18318735	*Escherichia coli*	JAUOMT01	298.67	117	5,062,605	50.55	177,514	Descending	GIEHV103	Non-CF	BBE	Anaerobic	Lumen
GP0377	GCA_030545795.1	SRS18318736	*Escherichia coli*	JAUOMS01	347.92	112	5,061,597	50.55	189,233	Descending	GIEHV103	Non-CF	BA + 100 μg/mL gentamicin	Anaerobic	Lumen
GP0381	GCA_030545785.1	SRS18318739	*Enterococcus faecalis*	JAUOMR01	540.05	46	2,989,852	37.27	222,605	Descending	GIEHV103	Non-CF	BA + 100 μg/mL gentamicin	Anaerobic	Mucosa
GP0382	GCA_030545765.1	SRS18318740	*Enterococcus faecalis*	JAUOMQ01	608.67	46	2,989,864	37.27	222,965	Descending	GIEHV103	Non-CF	BA + 100 μg/mL gentamicin	Anaerobic	Lumen
GP0395	GCA_030545695.1	SRS18318741	*Escherichia coli*	JAUOMP01	353.71	110	5,061,789	50.55	192,308	Descending	GIEHV103	Non-CF	BBE	Anaerobic	Lumen
GP0400	GCA_030545735.1	SRS18318742	*Escherichia coli*	JAUOMO01	230.16	112	5,026,146	50.57	203,814	Descending	GIEHV103	Non-CF	BBE	Anaerobic	Lumen
GP0409	GCA_030545685.1	SRS18318743	*Escherichia coli*	JAUOMN01	309.15	119	5,061,736	50.55	161,608	Descending	GIEHV103	Non-CF	BA + 100 μg/mL gentamicin	Anaerobic	Lumen
GP0410	GCA_030545665.1	SRS18318744	*Enterococcus faecalis*	JAUOMM01	560.68	47	2,990,117	37.27	195,960	Descending	GIEHV103	Non-CF	BA + 100 μg/mL gentamicin	Anaerobic	Lumen
GP0412	GCA_030545725.1	SRS18318745	*Enterococcus faecalis*	JAUOML01	584.57	47	2,989,464	37.27	222,965	Descending	GIEHV103	Non-CF	BA + 100 μg/mL gentamicin	Anaerobic	Lumen
GP0450	GCA_030545635.1	SRS18318746	*Enterococcus faecalis*	JAUOMK01	563.03	48	2,989,984	37.27	222,965	Ascending	GIEHV104	Non-CF	BA + 100 μg/mL gentamicin	Anaerobic	Lumen
GP0470	GCA_030545615.1	SRS18318747	*Streptococcus parasanguinis*	JAUOMJ01	896.56	35	2,139,427	41.69	92,998	Transverse	GIEHV104	Non-CF	BBE	Anaerobic	Lumen
GP0477	GCA_030545605.1	SRS18318748	*Streptococcus infantis*	JAUOMI01	769.81	17	1,768,524	39.31	263,788	Transverse	GIEHV104	Non-CF	BA + 100 μg/mL gentamicin	Anaerobic	Mucosa
GP0617	GCA_052978885.1	SRR35284286	*Enterococcus faecalis*	JBRKNS01	496.73	33	2,995,901	37.50	348,345	Ascending	GIECF103	CF	BA + 100 μg/mL gentamicin	Anaerobic	Lumen
GP0652	GCA_052978865.1	SRR35284285	*Enterococcus faecalis*	JBRKNR01	510.37	34	3,028,367	37.00	348,345	Ascending	GIECF103	CF	BA + 100 μg/mL gentamicin	Anaerobic	Mucosa
GP0688	GCA_052978845.1	SRR35284268	*Enterococcus faecalis*	JBRKNQ01	204.17	31	2,996,044	37.50	348,345	Transverse	GIECF103	CF	BA + 100 μg/mL gentamicin	Anaerobic	Lumen
GP0741	GCA_052978825.1	SRR35284269	*Escherichia coli*	JBRKNP01	293.49	93	5,094,755	50.50	356,950	Transverse	GIECF103	CF	McC	Aerobic	Mucosa
GP0759	GCA_052978805.1	SRR35284270	*Enterococcus faecalis*	JBRKNO01	263.14	33	2,993,956	37.50	348,345	Descending	GIECF103	CF	BA + 100 μg/mL gentamicin	Anaerobic	Lumen
GP0777	GCA_052978785.1	SRR35284271	*Escherichia coli*	JBRKNN01	202.43	89	5,094,512	50.50	418,134	Descending	GIECF103	CF	McC	Aerobic	Lumen
GP0796	GCA_052978765.1	SRR35284272	*Enterococcus faecalis*	JBRKNM01	348.76	38	2,993,949	37.50	283,605	Descending	GIECF103	CF	BA + 100 μg/mL gentamicin	Anaerobic	Mucosa
GP0813	GCA_052978745.1	SRR35284273	*Escherichia coli*	JBRKNL01	302.93	84	5,042,187	50.50	418,134	Descending	GIECF103	CF	McC	Aerobic	Mucosa
GP0831	GCA_052978725.1	SRR35284274	*Escherichia coli*	JBRKNK01	200.76	51	5,063,354	50.50	392,646	Transverse	GIECF105	CF	McC	Aerobic	Mucosa
GP0845	GCA_052978705.1	SRR35284275	*Escherichia coli*	JBRKNJ01	200.63	48	5,061,302	50.50	392,646	Descending	GIECF105	CF	McC	Aerobic	Lumen
GP0859	GCA_052978685.1	SRR35284284	*Escherichia coli*	JBRKNI01	235.65	50	5,061,883	50.50	392,646	Descending	GIECF105	CF	McC	Aerobic	Mucosa
GP0882	GCA_052978665.1	SRR35284283	*Enterococcus faecalis*	JBRKNH01	351.27	31	2,981,001	37.50	285,539	Descending	GIECF102	CF	BA + 100 μg/mL gentamicin	Anaerobic	Lumen
GP0924	GCA_052978645.1	SRR35284282	*Enterococcus faecalis*	JBRKNG01	402.82	29	3,014,041	37.50	398,541	Ascending	GIECF102	CF	BA + 100 μg/mL gentamicin	Anaerobic	Lumen
GP0925	GCA_052978625.1	SRR35284281	*Enterococcus faecalis*	JBRKNF01	398.93	28	3,013,322	37.50	398,541	Ascending	GIECF102	CF	BA + 100 μg/mL gentamicin	Anaerobic	Mucosa
GP0926	GCA_052978585.1	SRR35284280	*Enterococcus faecalis*	JBRKNE01	494.29	27	3,013,447	37.50	398,541	Transverse	GIECF102	CF	BA + 100 μg/mL gentamicin	Anaerobic	Lumen
GP0927	GCA_052978605.1	SRR35284279	Enterococcus faecalis	JBRKND01	399.31	29	3,014,031	37.50	398,547	Transverse	GIECF102	CF	BA + 100 μg/mL gentamicin	Anaerobic	Mucosa
GP0930	GCA_052978565.1	SRR35284278	Enterococcus faecalis	JBRKNC01	528.03	29	3,014,041	37.50	398,541	Descending	GIECF102	CF	BA + 100 μg/mL gentamicin	Anaerobic	Mucosa
GP0946	GCA_030545585.1	SRS18318750	*Clostridium perfringens*	JAUOMH01	522.93	36	3,350,141	28.04	450,303	Ascending	GIECF104	CF	BA + 100 μg/mL gentamicin	Anaerobic	Lumen
GP0982	GCA_030545565.1	SRS18318751	*Bacteroides thetaiotaomicron*	JAUOMG01	144.73	79	6,319,348	42.85	222,099	Ascending	GIEHV121	Non-CF	BA + 100 μg/mL gentamicin	Aerobic	Mucosa
GP0984	GCA_030545525.1	SRS18318752	*Bacteroides thetaiotaomicron*	JAUOMF01	231.94	54	6,254,654	42.78	310,843	Ascending	GIEHV121	Non-CF	BA + 100 μg/mL gentamicin	Anaerobic	Mucosa
GP0989	GCA_030541645.1	SRS18318753	*Thomasclavelia ramosa*	JAUNZI01	194.27	52	3,388,170	31.44	126,607	Ascending	GIEHV121	Non-CF	BBE	Anaerobic	Lumen
GP0992	GCA_030545535.1	SRS18318754	*Bacteroides thetaiotaomicron*	JAUOME01	294.56	53	6,240,291	42.86	270,363	Ascending	GIEHV121	Non-CF	BA + 100 μg/mL gentamicin	Anaerobic	Mucosa
GP1004	GCA_030545485.1	SRS18318755	*Lactococcus lactis*	JAUOMD01	714.95	50	2,406,846	34.79	201,476	Transverse	GIEHV121	Non-CF	BBE	Anaerobic	Lumen
GP1005	GCA_030541625.1	SRS18318756	*Thomasclavelia ramosa*	JAUNZH01	201.58	52	3,388,178	31.44	126,607	Transverse	GIEHV121	Non-CF	BBE	Anaerobic	Lumen
GP1008	GCA_030545495.1	SRS18318758	*Phocaeicola vulgatus*	JAUOMC01	336.77	114	4,716,152	42.17	129,202	Transverse	GIEHV121	Non-CF	BA + 100 μg/mL gentamicin	Anaerobic	Lumen
GP1009	GCA_030541605.1	SRS18318757	*Thomasclavelia ramosa*	JAUNZG01	203.31	54	3,387,905	31.44	125,352	Transverse	GIEHV121	Non-CF	BA + 100 μg/mL gentamicin	Anaerobic	Lumen
GP1018	GCA_030545445.1	SRS18318759	*Phocaeicola vulgatus*	JAUOMB01	359.79	135	4,770,123	42.16	161,246	Descending	GIEHV121	Non-CF	BA + 100 μg/mL gentamicin	Anaerobic	Lumen
GP1019	GCA_030545435.1	SRS18318761	*Phocaeicola vulgatus*	JAUOMA01	278.09	114	4,717,873	42.17	161,635	Descending	GIEHV121	Non-CF	BA + 100 μg/mL gentamicin	Anaerobic	Lumen
GP1028	GCA_030545425.1	SRS18318763	*Bacteroides thetaiotaomicron*	JAUOLZ01	162.14	70	6,267,143	42.79	278,301	Descending	GIEHV121	Non-CF	BA + 100 μg/mL gentamicin	Anaerobic	Mucosa
GP1029	GCA_030545385.1	SRS18318762	*Bacteroides thetaiotaomicron*	JAUOLY01	214.92	49	6,197,246	42.79	424,374	Descending	GIEHV121	Non-CF	BA + 100 μg/mL gentamicin	Anaerobic	Mucosa
GP1079	GCA_023274775.1	SRR35288057	*Bacteroides fragilis*	JALXKC01	629.00	27	5,223,116	43.26	491,737	Descending	GIEHZ100	Heterozygous	BBE + 100 μg/mL gentamicin	Anaerobic	Lumen
GP1080	GCA_023276265.1	SRR35288056	*Bacteroides fragilis*	JALXKG01	166.60	24	5,222,967	43.26	491,737	Descending	GIEHZ100	Heterozygous	BBE + 100 μg/mL gentamicin	Anaerobic	Mucosa
GP1081	GCA_023276305.1	SRR35288055	*Bacteroides fragilis*	JALXKI01	644.69	25	5,223,584	43.27	491,737	Descending	GIEHZ100	Heterozygous	BBE + 100 μg/mL gentamicin	Anaerobic	Lumen
GP1084	GCA_024055875.1	SRR35288054	*Clostridium perfringens*	JAMXLS01	62.93	19	3,374,914	28.12	987,734	Descending	GIEHZ100	Heterozygous	BBE	Anaerobic	Mucosa
GP1092	GCA_024295125.1	SRR35288051	*Streptococcus oralis*	JANCNM01	11.51	142	1,982,948	41.10	28,426	Descending	GIEHZ100	Heterozygous	BA + 100 μg/mL gentamicin	Aerobic	Mucosa
GP1097	GCA_024294865.1	SRS13657937	*Clostridium perfringens*	JANCNF01	503.90	20	3,374,224	28.11	2,112,524	Descending	GIEHZ100	Heterozygous	BA + 100 μg/mL gentamicin	Anaerobic	Mucosa
GP1101	GCA_024128825.1	SRR35288052	*Clostridium perfringens*	JAMYGY01	797.10	20	3,373,812	28.11	987,734	Descending	GIEHZ100	Heterozygous	BA + 100 μg/mL gentamicin	Anaerobic	Lumen
GP1115	GCA_024007805.1	SRR35288049	*Bacteroides fragilis*	JAMXKL01	548.10	29	5,226,328	43.26	446,345	Descending	GIEHZ100	Heterozygous	BBE + 100 μg/mL gentamicin	Anaerobic	Mucosa
GP1116	GCA_023274315.1	SRR35288048	*Bacteroides fragilis*	JALXJZ01	603.00	24	5,222,967	43.26	491,737	Descending	GIEHZ100	Heterozygous	BBE + 100 μg/mL gentamicin	Anaerobic	Lumen
GP1117	GCA_023274495.1	SRR35288047	*Bacteroides fragilis*	JALXKJ01	962.70	24	5,222,622	43.26	491,737	Descending	GIEHZ100	Heterozygous	BBE + 100 μg/mL gentamicin	Anaerobic	Lumen
GP1118	GCA_024296585.1	SRS13657871	*Bacteroides fragilis*	JANCNB01	515.45	23	5,222,840	43.26	491,737	Descending	GIEHZ100	Heterozygous	BBE + 100 μg/mL gentamicin	Anaerobic	Lumen
GP1125	GCA_024295065.1	SRR35288050	*Klebsiella pneumoniae*	JANCMY01	274.83	97	5,558,495	57.13	171,220	Descending	GIEHZ100	Heterozygous	BBE	Anaerobic	Mucosa
GP1134	GCA_023274755.1	SRR35288046	*Bacteroides fragilis*	JALXKD01	571.86	26	5,223,724	43.26	491,737	Descending	GIEHZ100	Heterozygous	BA + 100 μg/mL gentamicin	Anaerobic	Mucosa
GP1144	GCA_024137095.1	SRR35288043	*Klebsiella pneumoniae*	JAMYHZ01	720.56	95	5,558,472	57.13	185,845	Descending	GIEHZ100	Heterozygous	McC	Aerobic	Mucosa
GP1145	GCA_024295185.1	SRR35288041	*Klebsiella pneumoniae*	JANCMX01	665.13	58	5,555,205	57.14	402,419	Descending	GIEHZ100	Heterozygous	McC	Aerobic	Mucosa
GP1146	GCA_024295165.1	SRR35288040	*Klebsiella pneumoniae*	JANCNA01	562.33	60	5,537,560	57.15	391,040	Descending	GIEHZ100	Heterozygous	McC	Aerobic	Mucosa
GP1147	GCA_024296595.1	SRS13661004	*Klebsiella pneumoniae*	JANCNK01	464.75	55	5,551,790	57.14	391,040	Descending	GIEHZ100	Heterozygous	McC	Aerobic	Mucosa
GP1148	GCA_024294985.1	SRR35288045	*Klebsiella pneumoniae*	JANCNN01	858.89	60	5,539,094	57.14	402,419	Descending	GIEHZ100	Heterozygous	McC	Aerobic	Mucosa
GP1149	GCA_024128745.1	SRR35288044	*Klebsiella pneumoniae*	JAMYGX01	91.63	98	5,559,152	57.13	163,148	Descending	GIEHZ100	Heterozygous	McC	Aerobic	Mucosa
GP1163	GCA_052978545.1	SRR35284277	*Escherichia coli*	JBRKNB01	173.39	48	5,061,950	50.50	392,646	Ascending	GIECF105	CF	McC	Aerobic	Lumen
GP1176	GCA_052978525.1	SRR35284276	*Escherichia coli*	JBRKNA01	221.58	49	5,061,977	50.50	392,646	Ascending	GIECF105	CF	McC	Aerobic	Mucosa
GP1190	GCA_052978505.1	SRR35284267	*Escherichia coli*	JBRKMZ01	179.03	47	5,062,763	50.50	393,671	Transverse	GIECF105	CF	McC	Aerobic	Lumen

Colonoscopy samples were collected in accordance with approved institutional protocols (Dartmouth Health, IRB #07876). Bacterial isolates were obtained using blood agar (BA), *Bacteroides* bile esculin (BBE) agar, and MacConkey (McC) agar media by incubating both aerobically and anaerobically at 37°C for 48 h (6). Some media plates were supplemented with 100 μg/mL gentamicin as a selective agent. Colonies were purified by streaking three times. Genomic DNA was extracted from cell pellets using phenol–chloroform. Briefly, cells were lysed in buffer containing 0.5% SDS, 0.01 M EDTA, and 0.1 M LiCl, followed by sequential extraction with phenol:chloroform:isoamyl alcohol (25:24:1) and chloroform. DNA was precipitated with isopropanol and sodium acetate, washed with 70% ethanol, and resuspended in TE buffer. Sample libraries were prepared using the Illumina DNA Prep kit and IDT 10 bp UDI indices, and sequenced on an Illumina Novaseq 6000, producing 2 × 251 bp reads (UNH Hubbard Center for Genome Studies, Durham, NH, USA) and Illumina NextSeq 2000, producing 2 × 151 bp reads (SeqCenter, Pittsburgh, PA, USA, and SeqCoast Genomics, Portsmouth, NH, USA).

The project generated 360,914,139 raw reads, which were quality filtered using FastQC (v0.12) with default parameters (7). Assemblies were generated with SPAdes (v3.13) (8). Genome completeness and contamination were assessed with BUSCO (v5.3.2) and CheckM (v1.2.3) (9, 10). Taxonomic assignments were made with GTDB-Tk (v2.1.1) against the GTDB release r207 (11). Annotations were performed using the NCBI Prokaryotic Genome Annotation Pipeline (PGAP v6.8) and Prokka (v1.14.6) (12). Default parameters were used except where otherwise noted.

Across all isolates, genome sizes ranged from 1.77 to 6.32 Mb with GC contents of 28.0%–57.15%. Completeness was >99% for most assemblies, with contamination <1%. These genomes represent diverse gut-associated lineages, including members of the genera *Bacteroides*, *Clostridium*, *Escherichia*, *Enterococcu*s, *Lactococcus*, *Lactobacillus*, and *Thomasclavelia*, many of which have been implicated in CF-associated dysbiosis (4, 13). Some surprising isolates were from *Streptococcus*, which are generally considered oral microbes.

## Data Availability

Genome assemblies and raw sequencing reads have been deposited at NCBI under BioProject accession number PRJNA833080. Individual BioSample and GenBank accession numbers are provided in Table 1.
